# *Tropheryma whipplei* in Feces of Patients with Diarrhea in 3 Locations on Different Continents

**DOI:** 10.3201/eid2703.200182

**Published:** 2021-03

**Authors:** Gerhard E. Feurle, Verena Moos, Olfert Landt, Craig Corcoran, Udo Reischl, Matthias Maiwald

**Affiliations:** DRK Krankenhaus, Neuwied, Germany (G.E. Feurle);; Charité–Universitätsmedizin Berlin, Berlin, Germany (V. Moos);; TIB MOLBIOL Syntheselabor GmbH, Berlin, Germany (O. Landt);; National Reference Laboratory AMPATH, Centurion, South Africa (C. Corcoran);; Institute of Clinical Microbiology and Hygiene, University Hospital Regensburg, Regensburg, Germany (U. Reischl);; KK Women’s and Children’s Hospital, Singapore (M. Maiwald);; National University of Singapore, Singapore (M. Maiwald);; Duke-National University of Singapore Graduate Medical School, Singapore (M. Maiwald)

**Keywords:** *Tropheryma whipplei*, Whipple disease, diarrhea, gastroenteritis, South Africa, Singapore, Germany, enteropathogens, bacteria, enteric infections

## Abstract

We examined fecal specimens of patients with diarrhea from 3 continents for *Tropheryma whipplei* and enteropathogens. *T. whipplei* was most common in South Africa, followed by Singapore and Germany. Its presence was associated with the presence of other pathogens. An independent causative role in diarrhea appears unlikely.

*Tropheryma whipplei* is the causative agent of Whipple disease (*1*). The organism has also been detected in the feces of healthy or asymptomatic persons (*2*,*3*) and in the feces of patients with diarrhea (*4*–*6*). A causative role in gastroenteritis has been proposed.

To investigate the role of enteric *T. whipplei*, we examined fecal specimens of patients with diarrhea using conventional methods and PCR to detect enteric pathogens and *T. whipplei.* Our aim was to collect epidemiologic evidence regarding a causative role of *T. whipplei* in diarrhea.

## The Study

The 3 participating sites were the Molecular Biology Laboratory, AMPATH (Centurion, South Africa); the Department of Pathology and Laboratory Medicine at KK Women’s and Children’s Hospital (Singapore); and the Institute of Microbiology and Hygiene at the University Hospital Regensburg (Regensburg, Germany). We examined fecal samples from patients with diarrhea that were submitted for microbiological laboratory diagnosis; we used a combination of conventional tests and multiplex PCRs covering the pathogens shown in Table 1, with differences owing to local arrangements (Appendix).

**Table 1:** Frequency distribution of fecal pathogens in South Africa, Singapore, and Germany*

Location	No. (%)
Centurion, South Africa, 97 specimens	
* Tropheryma whipplei*	17 (17.53)
* Shigella* spp.	15 (15.46)
Rotavirus A	7 (7.22)
Adenovirus type F, 40, 41	5 (5.15)
* Salmonella* spp.	4 (4.12)
* Campylobacter* spp.	4 (4.12)
* Blastocystis hominis*	4 (4.12)
* Cryptosporidium* spp.	4 (4.12)
* Giardia lamblia*	4 (4.12)
* Yersinia enterocolitica*	1 (1.03)
* Escherichia coli*, EPEC, EHEC	1 (1.03)
* Aeromonas hydrophila*	1 (1.03)
* Plesiomonas shigelloides*	0
No infective agent detected	55 (56.70)
* T. whipplei* solo	8 (8.25)
Singapore, 193 specimens	
Rotavirus A	73 (37.82)
Norovirus GG1/2	35 (18.13)
* T. whipplei*	29 (15.03)
* Salmonella* spp.	24 (12.44)
* Campylobacter* spp.	17 (8.81)
* A. hydrophila*	10 (5.18)
Sapovirus	9 (4.66)
Astrovirus	8 (4.15)
Adenovirus type F, 40, 41	5 (2.59)
* G. lamblia*	2 (1.04)
* Dientamoeba fragilis*	2 (1.04)
* Shigella* spp.	1 (0.52)
* B. hominis*	1 (0.52)
* Vibrio* spp.	0
* Entamoeba histolytica*	0
* Y. enterocolitica*	0
* Cryptosporidium* spp.	0
No infective agent detected	55 (28.50)
* T. whipplei* solo	2 (1.04)
Regensburg, Germany, 300 specimens	
* Clostridioides difficile*	28 (9.33)
* T. whipplei*	10 (3.33)
* B. hominis*	10 (3.33)
* Campylobacter* spp.	8 (2.66)
* G. lamblia*	8 (2.66)
* Salmonella* spp.	3 (1.00)
* Y. enterocolitica*	2 (0.66)
* A. hydrophila*	2 (0.66)
* Shigella* spp.	1 (0.33)
* D. fragilis*	1 (0.33)
* Cryptosporidium* spp.	0)
* E. histolytica*	0
No infective agent detected	242 (80.66)
* T. whipplei* solo	7 (2.33)

*More than 1 pathogen was detected in some fecal specimens. *T. whipplei* solo indicates that *T. whipplei* was the sole organism detected. EPEC, enteropathogenic *Escherichia coli*; EHEC, enterohemorrhagic *Escherichia coli*.

We investigated a total of 590 fecal samples. In South Africa, 97 of 100 targeted samples were usable. In Singapore, 193 of 200 targeted specimens contained sufficient material; of these, 19 were originally submitted for bacterial culture, 77 for rotavirus antigen testing, and 97 for both. In Germany, we tested samples from 300 patients. In South Africa and Singapore, patients were mainly children, both outpatients and inpatients. In Singapore, the total included 13 immunocompromised children with hematologic/oncologic diseases and 1 with a short bowel syndrome. In Germany, all were inpatients and mostly elderly, about one quarter from the hematologic/oncologic ward (Figure).

**Figure:**
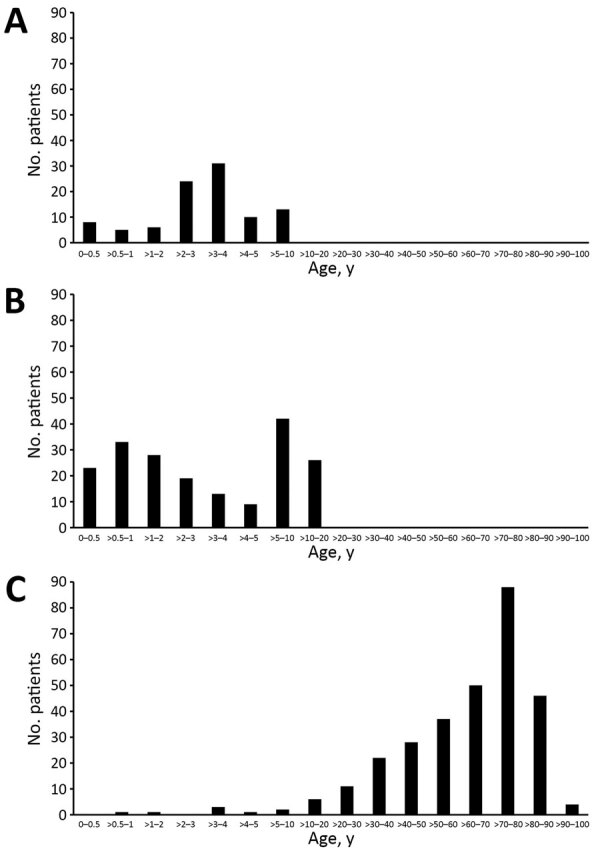
Age distribution of patients at 3 sites in study of *Tropheryma whipplei* in feces of patients with diarrhea: A) Centurion, South Africa; B) Singapore; and C) Regensburg, Germany.

Overall, 56 patients had positive test results for *T. whipplei* in the feces: 17 (17.5%) in South Africa, 29 (15%) in Singapore, and 10 (3.3%) in Germany. The frequency distribution of the organisms detected is shown in Table 1. In South Africa, *T. whipplei* was the most common fecal organism, followed by *Shigella*, rotavirus, and adenovirus. In Singapore, rotavirus was the most frequently detected organism, followed by norovirus, *T. whipplei*, and *Salmonella*. In Germany, *Clostridioides difficile* was the most frequently detected organism, followed by *T. whipplei* and *Blastocystis hominis*; viruses were not sought in Germany. The frequency of *C. difficile* likely reflects the high proportion of elderly inpatients.

Fecal specimens testing positive for *T. whipplei* averaged 0.91 other pathogens per specimen, in contrast to only 0.46 per specimen in those testing negative for *T. whipplei* (p = 0.0001; Table 2). Similarly, of the fecal specimens testing positive for *T. whipplei*, 69.6% contained other pathogens, in contrast to only 34.5% of the specimens testing negative for *T. whipplei* (p<0.0001; Appendix Table 1). Thus, specimens containing *T. whipplei* contained other pathogens about twice as frequently as specimens without *T. whipplei*. In Singapore, 1 specimen contained 4 pathogens: *T. whipplei*, *Blastocystis*, astrovirus, and *Dientamoeba*.

**Table 2:** Numbers of enteropathogens in fecal specimens with and without *Tropheryma whipplei* in South Africa, Singapore, and Germany*

Location	Specimens without *T. whipplei*			Specimens with *T. whipplei*	
	No. specimens	No. (rate) of enteropathogens		No. specimens	No. (rate) of enteropathogens
Centurion, South Africa	80	40 (0.50)		17	10 (0.59)
Singapore	164	145 (0.88)		29	38 (1.31)
Regensburg, Germany	290	60 (0.21)		10	3 (0.30)
Total†	534	245 (0.46)		56	51 (0.91)

*Total numbers and rates per specimen of all enteropathogens across all specimens collected at each site; multiple pathogens in a single specimen were counted as multiple entries. †Incidence rate χ^2^, p<0.0001.

Data on watery consistency and the presence of blood in feces were available for South Africa and Singapore, and microscopy data (e.g., erythrocytes, mucus, yeast cells) were available for South Africa (Appendix Table 2). There was no apparent relationship between these parameters and the presence of *T. whipplei*. Thus, an independent diarrheagenic role of *T. whipplei* was not apparent from these macroscopic and microscopic findings.

An association between the presence of *Campylobacter* and *T. whipplei* (Appendix Table 3) became apparent. Of 534 *T. whipplei*–negative fecal samples, 21 (3.9%) were positive for *Campylobacter* across all sites, whereas 8 (14.3%) of 56 *T. whipplei*–positive samples were positive for *Campylobacter*. This difference was statistically significant (p = 0.0035). In relative terms, specimens carrying *T. whipplei* contained *Campylobacter* 3 times more commonly than those without *T. whipplei*. When looking at the frequency ranking for all pathogens, *Campylobacter* rose from seventh position in *T. whipplei*–negative samples to being the fourth most common enteropathogen in *T. whipplei*–positive samples in South Africa and from fourth to second position in Singapore, whereas the position in Germany remained unchanged (Appendix Table 4).

The mechanisms underlying the *Campylobacter*–*Tropheryma* association remain unclear, but may include similar modes of acquisition. *T. whipplei* can be transmitted by the fecal–oral route (*7*,*8*). *Campylobacter* spp. are commensals in the gut of a variety of animals, especially poultry; the main infection routes for *Campylobacter* species are foodborne and fecal–oral transmission (*9*). Both *T. whipplei* and *Campylobacter* can be found in sewage (*9*–*11*).

Our study’s first limitation was that it was done in real-life settings of diagnostic laboratories where the routine investigations were supplemented by additional PCR tests (Appendix). Pathogens tested and diagnostic techniques differed among the 3 laboratories but were identical within each laboratory between the specimens with and without *T. whipplei*. However, this diversity may even increase the robustness of data. The proportions of fecal samples with no pathogen detected were 81% in Germany, 57% in South Africa, and 29% in Singapore. These findings reflect not only the absence of pathogens but also the pathogen spectrum investigated; a higher number of different pathogens investigated will lead to more positive results, and Singapore had the most comprehensive tests. A high rate of negative findings limits the analyses concerning co-infections of *T. whipplei* with other pathogens.

Second, our study did not include asymptomatic controls, as did the Global Enteric Multicenter Study (*12*,*13*). In South Africa, *T. whipplei* was the most frequent fecal microorganism, followed by *Shigella*, rotavirus, and adenovirus, in descending order, the last 3 exactly as in the Global Enteric Multicenter Study. In Singapore, *T. whipplei* was third after rotavirus and norovirus, but the ranking of rotavirus may be an artifact because rotavirus antigen was the most frequent ordered laboratory test.

We postulate that the different prevalence of pathogens at the 3 locations (Table 1) is related not just to the different diagnostic strategies but probably also to different climate, development, and hygiene. The Sustainable Development Goals indices for water, sanitation, and hygiene were 68, 66, and 90 in South Africa; 98, 99, and 97 in Singapore; and 100, 100, and 100 in Germany (*14*). These data reflect the order of prevalence of *T. whipplei* in feces in the 3 locations, which is in accordance with a prevalence approaching 50% in children in Laos (*15*).

## Conclusions

Using diagnostic specimens from microbiology laboratories on 3 continents, we were able to confirm that *T. whipplei* can be found frequently in the feces of patients with diarrhea (*4*,*5*). Across the 3 locations, the numbers of traditional enteropathogens were significantly increased in specimens also containing *T. whipplei*, and we found an association between the presence of *T. whipplei* and *Campylobacter*. Our findings support the hypothesis that enteric *T. whipplei* may not be causative for diarrhea but may possibly be a result of different sanitary and climatic conditions. 

Appendix Additional information on *Tropheryma whipplei* in feces of patients with diarrhea in 3 locations.
