# Supplementary material for: Tropheryma whipplei in Feces of Patients with Diarrhea in 3 Locations on Different Continents
Source: Emerg Infect Dis. 2021 Mar;27(3):932–5. doi: 10.3201/eid2703.200182 (PMC7920677; doi:10.3201/eid2703.200182)
Supplement: Appendix — Additional information on Tropheryma whipplei in feces of patients with diarrhea in 3 locations. [file 20-0182-Techapp-s1.pdf]

Article DOI: https://doi.org/10.3201/eid2703.200182

Tropheryma whipplei in Feces of Patients with Diarrhea in 3 Locations on Different Continents

Appendix

Methods

T. whipplei PCR testing was performed using the LightMix Modular Assay Kit *T. whipplei* (TIB Molbiol, https://www.tib-molbiol.com) (*1*), combined with the extraction control PhHV (TIB Molbiol), using LightCycler 480 instruments (Roche Molecular Diagnostics, https://diagnostics.roche.com), with determination of crossing point (Cp) values in positive samples. Multiplex PCR testing for other pathogens was performed using LightMix Modular Gastroenteritis Panel kits (TIB Molbiol), as reported for *Escherichia coli* (*2*), with varied pathogen composition.

All 3 sites used PCR to test for *T. whipplei*. Testing in Centurion, South Africa, included bacterial culture for *Salmonella* spp., *Shigella* spp., *Campylobacter* spp., *Yersinia enterocolitica*, *Aeromonas hydrophila*, *Plesiomonas shigelloides*, and *Vibrio* spp.; PCR for enteropathogenic *E. coli* (EPEC) and enterohemolytic *E. coli* (EHEC); and parasite microscopy and viral antigen testing (Coris BioConcept, https://www.corisbio.com) for rotavirus and adenovirus F (the latter 2 for children <5 years of age).

Testing in Singapore included routine culture (when requested) for *Salmonella* spp., *Shigella* spp., pathogenic *Campylobacter* spp., *Y. enterocolitica*, and *Vibrio* spp., and antigen testing (when requested) for rotavirus A. Multiplex PCR was done for *Salmonella* spp., *Shigella* spp., pathogenic *Campylobacter* spp., *Y. enterocolitica, A. hydrophila*, rotavirus A, adenovirus type F, astrovirus, norovirus genogroups I and II, sapovirus, *Blastocystis hominis*, *Cryptosporidium* spp., *Dientamoeba fragilis, Entamoeba histolytica*, and *Giardia lamblia*. Fecal bacterial culture or rotavirus antigen testing was done in subsets of samples based on physician

requests; all fecal specimens were tested by multiplex PCR. Culture for *Y. enterocolitica* was performed for bloody feces, culture for *Vibrio* spp. for watery feces.

Testing in Regensburg, Germany, included PCR for *Salmonella* spp., *Shigella* spp., pathogenic *Campylobacter* spp., *Y. enterocolitica*, *A. hydrophila*, *Clostridioides difficile*, *B. hominis*, *D. fragilis*, and *G. lamblia*. Because of specific arrangements in Regensburg, fecal samples were anonymized before PCR testing and culture results made unavailable; viral pathogen testing was not done. At all 3 sites, any positive findings were included in the evaluation of the results, regardless of the method by which they were obtained.

All samples that were positive for *T. whipplei* in Centurion and in Regensburg were retested in an independent, previously validated PCR assay targeting the *rpoB* gene of *T. whipplei* (*3*); in Singapore, the nucleic acid extracts were exhausted during prior rounds of testing and were unavailable for retesting.

In an extension of the project, 20 fresh half chickens were purchased at 13 wet markets in Singapore. Skin swabs from each animal were obtained using flocked swabs (FLOQSwabs, https://www.copanusa.com). DNA was extracted from the swabs using the QIAGEN PC purification kit (QIAGEN, https://www.qiagen.com) and subjected to the TIB Molbiol PCRs for *Campylobacter* spp. and *T. whipplei*.

Comparisons between groups on frequency counts (proportions) were done using Fisher exact test, those on incidence rates using a χ^2 test, and those involving age using a 2-sample *t*-test. Statistical significance was set at $p<0.05$.

Results

There were 303 (51.4%) male and 287 (48.6%) female patients in the study. The percentage of males was 60.7% among those with specimens positive for *T. whipplei* and 50.4% among those with specimens negative for *T. whipplei*, but this was not significant (Fisher exact test; p = 0.16). The mean age in South Africa was 3.2 years (3.12 and 3.57 years for *T. whipplei*-negative and -positive patients, respectively), in Singapore it was 5.04 years (4.98 and 5.38 years, respectively), and in Germany it was 62.41 years (62.41 and 62.50 years, respectively),

with no significant age differences between *T. whipplei* negative and positive patients within each study site (p = 0.327, 0.674, and 0.989, respectively; 2-sample *t*-test).

Retesting of the nucleic acid extracts from Centurion and from Regensburg with the *rpoB* gene PCR for *T. whipplei* revealed 2 positive TIB Molbiol PCR results for *T. whipplei* with Cp values of 37 and 33.6 in Centurion and 3 positive results with Cp values of 36.8, 36.88, and 39.17 in Regensburg that were not confirmed by the *rpoB* gene PCR. However, even if these specimens were assumed negative, this would not affect the overall results.

The PCR results in swabs of chicken skin in Singapore were positive for *Campylobacter* spp. in 10 of 20 chickens, with Cp values of 35.8 ± 2.46 (mean ± standard deviation). All test results for *T. whipplei* in chicken skin were negative.

References

1. Frickmann H, Hanke M, Hahn A, Schwarz NG, Landt O, Moter A, et al. Detection of *Tropheryma whipplei* in stool samples by one commercial and two in-house real-time PCR assays. Trop Med Int Health. 2019;24:101–8. PubMed https://doi.org/10.1111/tmi.13172

2. Hahn A, Luetgehetmann M, Landt O, Schwarz NG, Frickmann H. Comparison of one commercial and two in-house TaqMan multiplex real-time PCR assays for detection of enteropathogenic, enterotoxigenic and enteroaggregative *Escherichia coli*. Trop Med Int Health. 2017;22:1371–6. PubMed https://doi.org/10.1111/tmi.12976

3. Moter A, Schmiedel D, Petrich A, Wiessner A, Kikhney J, Schneider T, et al. Validation of an rpoB gene PCR assay for detection of *Tropheryma whipplei*: 10 years' experience in a National Reference Laboratory. J Clin Microbiol. 2013;51:3858–61. PubMed https://doi.org/10.1128/JCM.01703-13

Appendix Table 1. Numbers of specimens with any enteropathogens in specimens without and with *T. whipplei*

	Specimens without *T. whipplei*		Specimens with *T. whipplei*	
Location	No. specimens	No. (%) of specimens with enteropathogens*	No. specimens	No. (%) of specimens with enteropathogens*
Centurion, South Africa	80	28 (35.0)	17	9 (52.9)
Singapore	164	109 (66.5)	29	27 (93.1)
Regensburg, Germany	290	47 (16.2)	10	3 (30.0)
Total†	534	184 (34.5)	56	39 (69.6)

*Numbers and percentages of specimens that contained any other pathogens, regardless of number of pathogens in a given specimen.
†Fisher exact test, p<0.0001.

Appendix Table 2. Macroscopic and microscopic findings in the fecal specimens without and with *T. whipplei* in Centurion and Singapore

South Africa	Specimens without *T. whipplei* (80 specimens)		Specimens with *T. whipplei* (17 specimens)	
Finding	n	%	n	%
Watery	41	51.3	8	47.1
Erythrocytes	21	26.3	7	41.2
Mucus	32	40.0	8	47.1
Pus cells	60	75.0	12	70.5
Charcot-Leyden crystals	4	5.0	1	5.9
Oil droplets	8	10.0	0	0
Yeast cells	30	37.5	5	29.4
**Singapore**	**Specimens without *T. whipplei* (164 specimens)**		**Specimens with *T. whipplei* (29 specimens)**	
Finding	n	%	n	%
Watery	24	14.6	2	6.9
Bloody	8	4.9	2	6.9

Appendix Table 3. Frequency of *Campylobacter* spp. and *T. whipplei* detected in the feces of patients with diarrhea

	Samples without *T. whipplei*		Samples with *T. whipplei*	
Location	No. specimens	No. (%) of specimens with *Campylobacter* spp.	No. specimens	No. (%) of specimens with *Campylobacter* spp.
Centurion, South Africa	80	3 (3.8)	17	1 (5.9)
Singapore	164	10 (6.1)	29	7 (24.1)
Regensburg, Germany	290	8 (2.8)	10	0 (0)
Total*	534	21 (3.9)	56	8 (14.3)

* Fisher exact test, p = 0.0035.

Appendix Table 4. Frequency ranking of fecal enteropathogens in specimens without and with *T. whipplei*

Rank		n	%	Rank		n	**%**
South Africa							
Without *T. whipplei* (80 patients)				With *T. whipplei* (17 patients)			
1	*Shigella* spp.	10	12.5	1	*Shigella* spp.	5	29.4
2	Rotavirus A	5	6.3	2	Rotavirus A	2	11.8
2	Adenovirus type F (41, 42)	5	6.3	2	*Blastocystis*	2	11.8
4	*Salmonella* spp.	4	5	4	*Campylobacter* spp.*	1	5.9
4	*Cryptosporidium*	4	5	5	*Yersinia enterocolitica*	0	0
4	*Giardia lamblia*	4	5	5	*E. coli* EPEC, EHEC†	0	0
7	*Campylobacter* spp.	3	3.8	5	*Cryptosporidium*	0	0
8	*Blastocystis*	2	2.5	5	*Giardia lamblia*	0	0
9	*E. coli* EPEC, EHEC	1	1.3	5	Adenovirus type F (41, 42)	0	0
9	*Aeromonas hydrophila*	1	1.3	5	*Aeromonas hydrophila*	0	0
9	*Yersinia enterocolitica*	1	1.3	5	*Salmonella* spp.	0	0
	No enteropathogen detected	47	58.8		No enteropathogen detected	8	47.1
Singapore							
Without *T. whipplei* (164 patients)				With *Tropheryma whipplei* (29 patients)			
1	Rotavirus A	59	36.0	1	Rotavirus A	14	48.3
2	Norovirus GG1/2	29	17.7	2	*Campylobacter spp.*	7	24.1
3	*Salmonella* spp.	21	12.8	3	Norovirus GG1/2	6	20.7
4	*Aeromonas hydrophila*	10	6.1	4	*Salmonella* spp.	3	10.3
4	*Campylobacter* spp.	10	6.1	4	Sapovirus	3	10.3
6	Sapovirus	6	3.7	6	Astrovirus	2	6.9
6	Astrovirus	6	3.7	7	*Giardia lamblia*	1	3.4
8	Adenovirus type F (41, 42)	5	3.0	7	*Dientamoeba fragilis*	1	3.4
9	*Giardia lamblia*	1	0.6	7	*Blastocystis hominis*	1	3.4
9	*Dientamoeba fragilis*	1	0.6	10	*Cryptosporidium*	0	0
9	*Shigella* spp.	1	0.6	10	Adenovirus type F (41, 42)	0	0
12	*Vibrio* spp.	0	0	10	*Shigella* spp.	0	0
12	*Blastocytis hominis*	0	0	10	*Aeromonas hydrophila*	0	0
12	*Entamoeba histolytica*	0	0	10	*Entamoeba histolytica*	0	0
12	*Yersinia enterocolitica*	0	0	10	*Yersinia enterocolitica*	0	0
12	*Cryptosporidium*	0	0	10	*Vibrio* spp.	0	0
	No enteropathogen detected	52	31.7		No enteropathogen detected	2	6.9
Germany							
Without *T. whipplei* (290 patients)				With *T. whipplei* (10 patients)			
1	*Clostridioides difficile*	26	9.0	1	*Clostridioides difficile*	2	20
2	*Blastocystis hominis*	10	3.5	2	*Giardia lamblia*	1	10
3	*Campylobacter* spp.	8	2.8	3	*Campylobacter* spp.	0	0
4	*Giardia lamblia*	7	2.4	3	*Blastocystis hominis*	0	0

Rank		n	%	Rank		n	%
5	*Salmonella* spp.	3	1.3	3	*Salmonella* spp.	0	0
6	*Aeromonas hydrophila*	2	0.7	3	*Aeromonas hydrophila*	0	0
6	*Yersinia enterocolitica*	2	0.7	3	*Yersinia enterocolitica*	0	0
8	*Shigella* spp.	1	0.3	3	*Shigella* spp.	0	0
8	*Dientamoeba*	1	0.3	3	*Dientamoeba*	0	0
10	*Cryptosporidium*	0	0	3	*Cryptosporidium*	0	0
10	*Entamoeba histolytica*	0	0	3	*Entamoeba histolytica*	0	0
	No enteropathogen detected	235	81.0		No enteropathogen detected	7	70
	Total specimens analyzed	534			Total specimens analyzed	56	
	Total specimens without enteropathogen	334	62.5		Total specimens without enteropathogen	17	30.4
	Total specimens with enteropathogens	200	37.5		Total specimens with enteropathogens	39	69.6

**Campylobacter spp.* is shaded to highlight the changing rank.
†EPEC, enteropathogenic *Escherichia coli*; EHEC, enterohemorrhagic *Escherichia coli*.
